# AGE-FRIENDLY COMMUNITIES IN THE ANDES: CONSIDERATIONS TO IMPROVE UPTAKE

**DOI:** 10.1093/geroni/igad104.2358

**Published:** 2023-12-21

**Authors:** Jonathan Guillemot, Mildred Warner

**Affiliations:** Universidad San Francisco de Quito, Quito, Pichincha, Ecuador; Cornell University, Ithaca, New York, United States

## Abstract

Despite the demographic aging of the Global South, the uptake of WHO’s age-friendly cities framework remains extremely low. The Andean region of Latin America is currently represented by only four cities, out of more than 1,400 globally. Causes may include the partial inadequacy of the eight WHO domains for communities in the Andean region. We argue for a broader human ecological framework to address the macro, meso and micro levels to better address the context, challenges and opportunities for age-friendly cities in the Andean region. WHO’s age-friendly city domains are focused primarily at the meso (community) scale – on built environment, services and participation. We call for more attention to the macro policy scale, in societies where robust social security systems cannot be assumed. We also call for more attention to the micro scale, to recognize the critical role of family and informal care supports. We question whether the WHO domains are the result of a design bias, with Global North settings in mind for its development. We find the domains of UNICEF’s child-friendly cities initiative, which give more attention to the realities of the Global South, helpful to broaden WHO’s age friendly framework.

